# 3-month oral nutritional supplementation adherence impacts positively on survival in malnourished older patients following hip fracture: a real-life study

**DOI:** 10.3389/fnut.2026.1757193

**Published:** 2026-03-11

**Authors:** Francisco José Sánchez-Torralvo, Verónica Pérez del Río, Luis Ignacio Navas Vela, María García-Olivares, Nuria Porras, Jose Abuín Fernández, Gabriel Olveira

**Affiliations:** 1 Unidad de Gestión Clínica de Endocrinología y Nutrición, Hospital Regional Universitario de Málaga, Malaga, Spain; 2 IBIMA Plataforma BIONAND, Instituto de Investigación Biomédica de Málaga, Málaga, Spain; 3 Departamento de Medicina y Dermatología, Facultad de Medicina, University of Malaga, Malaga, Spain; 4 Unidad de Gestión Clínica de Cirugía Ortopédica y Traumatología, Hospital Regional Universitario de Málaga, Malaga, Spain; 5 Unidad de Gestión Clínica de Endocrinología y Nutrición, Hospital Universitario Torrecárdenas, Almeria, Spain; 6 Centro de Investigación Biomédica en Red de Diabetes y Enfermedades Metabólicas Asociadas (CIBERDEM), Instituto de Salud Carlos III, Malaga, Spain

**Keywords:** adherence, aged, hip fractures, mortality, oral nutritional supplement

## Abstract

**Introduction:**

Several factors influence mortality and survival after hip fracture, including nutritional status, which is associated with both incidence and prognosis. However, there is little evidence on the impact of oral nutritional supplements (ONS) on the survival of these patients, and the available results are mixed. Our aim was to analyze the effect of adherence to ONS treatment on post-hospital mortality, with the hypothesis that this would be lower in an adherent group.

**Methods:**

Prospective study of patients aged 65 years or older, admitted for fragility hip fracture. Follow-up was carried out at 3, 6 and 12 months to evaluate retrieval of ONS in pharmacies and survival. Adherence was considered if ONS were withdrawn for 3 months or longer. The sample was divided into four groups: (1) well-nourished patients without prescription of ONS, (2) moderately malnourished patients without prescription of ONS, (3) moderate or severely malnourished patients with prescription of ONS but without adherence, and (4) moderately or severely malnourished patients with prescription of ONS and adherence. Mortality between groups was compared by means of a Cox regression, adjusted for confounding variables.

**Results:**

300 patients were included (mean age 82.9 years; 79.3% female), with severe malnutrition in 19.7%. Non-adherent malnourished patients showed a significantly higher risk of death than adherent malnourished patients (HR = 3.67; 95% CI: 1.41–9.57; *p* = 0.008). Non-adherent malnourished patients had a significantly higher risk of death compared to well-nourished patients (HR = 2.95; 95% CI: 1.31–6.65; *p* = 0.009). Malnourished patients without ONS had a non-significant higher mortality risk than well-nourished patients (HR = 1.66; 95% CI: 0.72–3.84, *p* = 0.236). Adherent malnourished patients showed a non-significant trend toward lower mortality than well-nourished patients (HR = 0.80; 95% CI: 0.25–2.56; *p* = 0.712).

**Conclusion:**

In our study, 3-month adherence to ONS is associated with a reduction in 3, 6 and 12-month mortality in older patients with a hip fracture when compared to non-adherent patients and shows a trend toward an improved survival rate than that of well-nourished patients.

## Introduction

The development of strategies for the prevention and management of fragility fractures is an essential response to the rising incidence of osteoporosis in the older population. These fractures, caused by low-impact trauma, predominantly affect the humerus, wrist, vertebrae, and hip (1), with hip fractures being particularly associated with significant risks of mortality and refracture, leading to substantial economic costs (2, 3). Annually, there are approximately 1.7 million cases of hip fractures globally (4), with 620,000 occurring in Europe (5). Multiple factors have been associated with mortality and survival in patients following hip fracture. Overall mortality following hip fracture has declined over the past six decades. This improvement is likely attributable to advances in multidisciplinary care strategies that integrate both prevention and tailored treatment approaches, addressing the broad spectrum of patient-specific variables (6). Functional independence appears to be positively influenced by osteoporosis prophylaxis with calcium and vitamin D (7), as well as by pharmacological treatment for osteoporosis (8). In contrast, poor nutritional status has been linked to worse functional outcomes (7, 9, 10).

Fracture Coordination Units (FCUs), also known as Fracture Liaison Services (FLS), adopt a multidisciplinary strategy to promote secondary prevention of fragility fractures (11). Since their implementation in 2011, these programs have demonstrated significant efficacy in reducing all-cause mortality among patients with hip fractures (12, 13). Nutrition plays a pivotal role within this framework, as a strong association has been identified between malnutrition and both the incidence and prognosis of hip fractures (14). Malnutrition has consistently been linked to a greater risk of complications, impaired functional recovery, and increased mortality rates (7, 13, 15–21). Malnutrition is highly prevalent among older individuals admitted with hip fractures, and the condition frequently persists for several months after injury. This chronic undernutrition is driven by a combination of insufficient dietary intake to meet increased metabolic demands, systemic inflammation, age-related anorexia, immobility, and the presence of comorbidities (16). Importantly, nutritional interventions have demonstrated effectiveness in improving both nutritional status and functional outcomes in older adults recovering from hip fracture (16). Nevertheless, there are not many works that analyze their impact on mortality.

In previous works we demonstrated that malnutrition was associated with mortality in patients with a fragility hip fracture (22, 23). Our hypothesis for the current study is that mortality may be lower among patients with malnutrition with a hip fracture who are adherent to the prescribed oral nutritional supplement (ONS) compared to those who do not. The objective of this work is to examine mortality outcomes associated with adherence to ONS by leveraging data from a well-characterized cohort.

## Materials and methods

**Figure 1:**
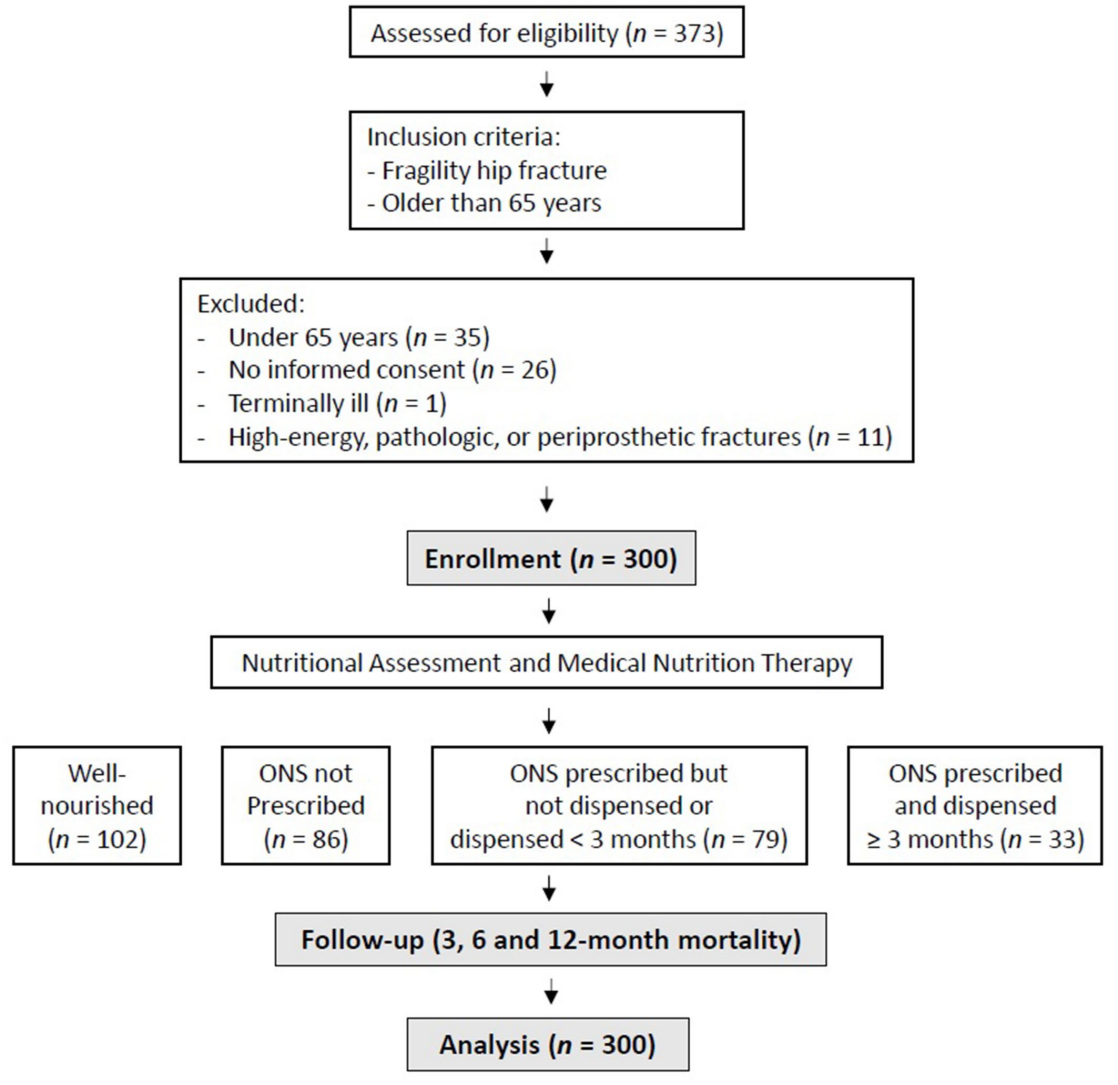
Study flow diagram.

This cohort study was conducted on patients over 65 years of age admitted for fragility hip fractures at a tertiary care hospital between September 2019 and February 2021 (22, 23). For this study, fragility hip fracture was defined as a proximal femur fracture resulting from low-energy trauma, such as a fall from standing height or less. According to the study protocol, a total of 11 patients were excluded due to high-energy, pathologic (e.g., malignancy-related, Paget disease, osteomalacia), or periprosthetic fractures and 1 patient was excluded due to terminal illness (22, 23). The study design is illustrated in Figure 1.

### Medical and functional assessments

The type of fracture and the presence of previous fractures were recorded. Medical comorbidities were measured using the Charlson Comorbidity Index (CCI), which assigns a score that increases with the 10-year risk of death based on a patient’s comorbid diseases (24). Pre-fracture functional status was assessed using the Barthel Index (BI), which measures a person’s ability to care for themselves (ranging from 0 to 100, with 100 indicating an independent person) (25), and the Functional Ambulation Category Scale (FAC), which measures the level of manual assistance required for ambulation (ranging from 0 to 5, with 5 indicating an independent ambulator) (26).

### Anthropometry and nutritional assessment

Nutritional status was assessed within the first 24–48 h post-surgery using Subjective Global Assessment (SGA), since it is a validated tool for assessing malnutrition in hospitalized older adults (27, 28) that has been associated to higher mortality in patients with a hip fracture (17) and is routinely used in our Nutrition Unit as part of standard clinical practice. It integrates medical history (recent weight and intake changes, gastrointestinal symptoms, and functional capacity) with a focused physical examination, allowing reliable nutritional assessment in acute settings such as hip fracture, where objective anthropometric measurements are frequently limited. Alternative screening tools such as MNA or MNA-SF were not systematically collected and were not prespecified in the study protocol. When possible, height was measured using a stadiometer (Holtain Limited, Crymych, United Kingdom) and body mass was measured using a scale set to 0.1 kg (SECA 665, Hamburg, Germany). When height and body mass could not be determined, patient-reported data were used. Body mass index (BMI) was calculated, and calf circumference was measured.

### Treatment

ONS were prescribed to patients diagnosed with either severe malnutrition or moderate malnutrition with some complication or a reported decreased oral intake according to usual clinical practice based on SGA. In most of them, a high-energy, high-protein ONS (HC-HP ONS) enriched with *β*-hydroxy-β-methylbutyrate (HMB) was used. In patients with diabetes mellitus and suboptimal glycemic control or hyperglycemia, a diabetes-specific ONS with a modified carbohydrate profile and lower glycemic index was prescribed. In patients with chronic kidney disease not requiring dialysis, a renal-specific ONS formulation with reduced protein, potassium, and phosphorus content was used. Patients with moderate malnutrition who were not prescribed ONS were given general diet recommendations to increase their energy and protein intake. In our setting, the national health system provides universal coverage, and oral nutritional supplements are subsidized medical foods. While specific individual socioeconomic indicators were not systematically recorded, the universal nature of the healthcare system minimizes disparities in access to surgery and post-discharge nutritional therapy.

### Follow-up

Patients were followed clinically 3 months post-discharge. Subsequently, they were followed telematically at 6 and 12 months by reviewing their medical history at the hospital software database Diraya. By accessing its prescription software, we were able to check whether and when patients had retrieved ONS from the pharmacy. ONS adherence was determined from pharmacy records, considering retrieval for 3 months or more as adherent and less than 3 months, including those who failed to collect them from the pharmacy, as non-adherent. In our healthcare system, electronic pharmacy records serve as the sole official source for ONS distribution once the patient is discharged. This method allows for a real-world effectiveness analysis using validated administrative data, providing a pragmatic assessment of treatment persistence in the elderly population. Mortality, the primary outcome, was recorded 3, 6 and 12 months after discharge.

### Statistical analysis

Data analysis was performed using SPSS 22.0 (SPSS Inc., Chicago, IL, United States, 2013). Qualitative variables were expressed as percentages. Quantitative variables were expressed as mean ± standard deviation. Kaplan–Meier survival curves and multivariable Cox proportional hazards regression were utilized to evaluate the association between nutritional status, ONS use and 3, 6 and 12-month mortality. Patients were classified into four groups: (1) well-nourished individuals without prescription of ONS, (2) moderately malnourished patients without prescription of ONS, (3) moderate or severely malnourished patients with prescription of ONS but who did not retrieve them from pharmacies or retrieved them for shorter than 3 months (non-adherent) and (4) moderate or severely malnourished patients with prescription of ONS who retrieved them for 3 months or longer (adherent). No formal *a priori* sample size calculation was performed; analyses should be interpreted as exploratory within a real-world cohort.

The Cox model was adjusted for relevant clinical covariates, including sex, age, CCI, BI and FAC. The proportional hazards assumption was assessed graphically using log-minus-log plots. From the fitted Cox model, adjusted survival curves were derived to visually compare the estimated survival probabilities across the four groups, controlling for the effects of the covariates. These adjusted curves reflect survival estimates under the average distribution of the covariates in the study population. Although fracture subtype was recorded, it was not included in the final adjustment model. Specifically, subtrochanteric fractures represented only 6.4% of the cohort, and their inclusion would have limited the model’s statistical robustness. To reduce potential immortal-time bias related to ONS adherence classification, a sensitivity analysis was performed excluding patients who died within the first 30 days after hip fracture surgery. This sensitivity analysis was restricted to malnourished patients with ONS prescribed, as adherence cannot be defined in the absence of prescription. A conservative 3-month landmark analysis was additionally performed, including only patients alive at 90 days.

## Results

A total of 300 patients were included (Figure 1). The general characteristics of the sample are shown in Table 1. The study population showed a clear female predominance, with 238 women (79.3%) and 62 men (20.7%). Regarding the baseline nutritional assessment performed via SGA, 102 patients (34%) were classified as well-nourished, while 198 patients (66%) presented some degree of malnutrition, with 139 (46.3%) categorized as moderately malnourished and 59 (19.7%) as severely malnourished. Baseline objective measurements, including detailed anthropometry, bioelectrical impedance analysis (BIA), and handgrip strength, are summarized in Table 1. Table 2 shows the characteristics of the four groups of patients, including mortality at 3, 6 and 12 months after surgery. Overall mortality rate at 12 months varied significantly according to nutritional status and the management of ONS. Among well-nourished patients, the one-year mortality rate was 8.8%. In the malnourished group, outcomes differed based on ONS prescription and dispensing: patients who were not prescribed ONS had a mortality rate of 26.7%, whereas those with an ONS prescription showed markedly different survival figures depending on adherence. Specifically, malnourished patients who were adherent to ONS exhibited a mortality rate of 15.2%, while those classified as non-adherent reached a mortality rate of 36.7% at the end of the 12-month follow-up. Crude ONS adherence rates (<3 months vs. ≥ 3 months) according to baseline functional status (Barthel Index categories) and comorbidity burden (Charlson Comorbidity Index categories) are shown in Supplementary Tables 1, 2.

**Table 1:** General features and nutritional assessment.

**Number of patients**	**n = 300**
**Age (years)**	mean ± SD
	82.9 ± 7.1
**Gender**	mean ± SD
Male	62 (20.7)
Female	238 (79.3)
**BMI (kg/m** ^**2**^ **)**	mean ± SD
Male	25.9 ± 3.5
Female	25.8 ± 3.4
**Triceps skinfold (mm)**	mean ± SD
Male	11.9 ± 4
Female	15.7 ± 6.1
**Calf circumference (cm)**	mean ± SD
Male	32.4 ± 2.8
Female	30.7 ± 3.8
**Fat-free mass (anthropometry) (kg)**	mean ± SD
Male	53.4 ± 8.3
Female	42.8 ± 7.3
**FFMI (anthropometry) (kg/m2)**	mean ± SD
Male	19.4 ± 8.6
Female	17.5 ± 2.1
**Phase angle (°)**	mean ± SD
Male	5.18 ± 1.13
Female	4.5 ± 0.94
**Fat-free mass (BIA) (kg)**	mean ± SD
Male	57.6 ± 7.8
Female	42.9 ± 5.4
**FFMI (BIA) (kg/m** ^**2**^ **)**	mean ± SD
Male	20.9 ± 9.6
Female	15.4 ± 1.5
**Handgrip strength (kg)**	mean ± SD
Male	19.7 ± 9.7
Female	7.7 ± 6.4
**SGA**	n (%)
Well-nourished	102 (34)
Moderate malnutrition or at risk	139 (46.3)
Severe malnutrion	59 (19.7)

Abbreviations: BMI=Body Mass Index; FFMI = fat-free mass index; BIA = Bioelectrical Impedance Analysis; SGA=Subjective Global Assessment; SD=Standard Deviation. Cualitative variables are expressed as n (%). Quantitative variables are expressed as mean ± SD.

**Table 2:** Features of patients classified according to nutritional assessment and ONS prescription.

	Normo-nourished	Moderately malnourished without ONS prescribed	Moderately or severely malnourished with ONS prescribed but not dispensed or dispensed < 3 months	Moderately or severely malnourished with ONS prescribed and dispensed > 3 months	*p* value
**Number of patients**	**n= 102**	**n= 86**	**n= 79**	**n= 33**	
**Age (years)**	mean ± SD	mean ± SD	mean ± SD	mean ± SD	p value
	79.9 ± 7.8*	84.4 ± 6†	83.8 ± 6.9	85.5 ± 7.1†	< 0.001
**Sex**	n (%)	n (%)	n (%)	n (%)	p value
Men	33 (32.3)*	16 (18.6)	10 (12.6)	3 (9.1)	0.02
Women	69 (67.6)*	70 (81.4)	69 (83.4)	30 (90.9)	
**Charlson Index**	mean ± SD	mean ± SD	mean ± SD	mean ± SD	p value
	4.75 ± 1.73*	6.1 ± 1.65	5.97 ± 1.92	6.67 ± 1.9	< 0.001
**Barthel Index**	mean ± SD	mean ± SD	mean ± SD	mean ± SD	p value
	87.4 ± 19.9*	60.1 ± 27.6†	73.4 ± 28.8	67.4 ± 27.4†	< 0.001
**FAC Scale**	n (%)	n (%)	n (%)	n (%)	p value
0	51 (50)*	10 (11.6)	12 (15.2)	4 (12.1)	< 0.001
1,2,3	49 (48)*	118 (80.2)	62 (78.5)	27 (81.8)	
4,5	2 (1.9)*	7 (8.1)	5 (6.3)	2 (6)	
**Type of fracture**	n (%)	n (%)	n (%)	n (%)	p value
Pertrochanteric	44 (43.1)	38 (44.2)	34 (43)	19 (57.6)	0.33
Subcapital	45 (44.1)	37 (43)	37 (46.8)	12 (36.4)	
Subtronchanteric	9 (8.8)	6 (6.9)	3 (3.8)	0 (0)	
Basicervical	4 (3.9)	5 (5.8)	5 (6.3)	2 (6)	
**Length of stay**	n (%)	n (%)	n (%)	n (%)	p value
	7.8 ± 5.5	7.6 ± 3.4	9.2 ± 7.3	8 ± 4.2	0.19
**3-month exitus**	n (%)	n (%)	n (%)	n (%)	p value
	3 (2.9) ‡	12 (13.9)	15 (18.9) ‡§	0 (0) §	< 0.001
**6-month exitus**	n (%)	n (%)	n (%)	n (%)	p value
	6 (5.9) ‡	17 (19.8)	23 (29.1) ‡§	3 (9) §	< 0.001
**12-month exitus**	n (%)	n (%)	n (%)	n (%)	p value
	9 (8.8) ‡	23 (26.7)	29 (36.7) ‡§	5 (15.2) §	< 0.001

Abbreviations: SD=Standard Deviation; FAC= Functional Ambulation Category.

*: *p* < 0.05 vs. all other groups; †: *p* < 0.05 between no ONS and ONS ≥3 months; ‡*p* < 0.05 between normo-nourished and ONS <3 months; §*p* < 0.05 between ONS <3 months and ONS ≥3 months.

**Figure 2:**
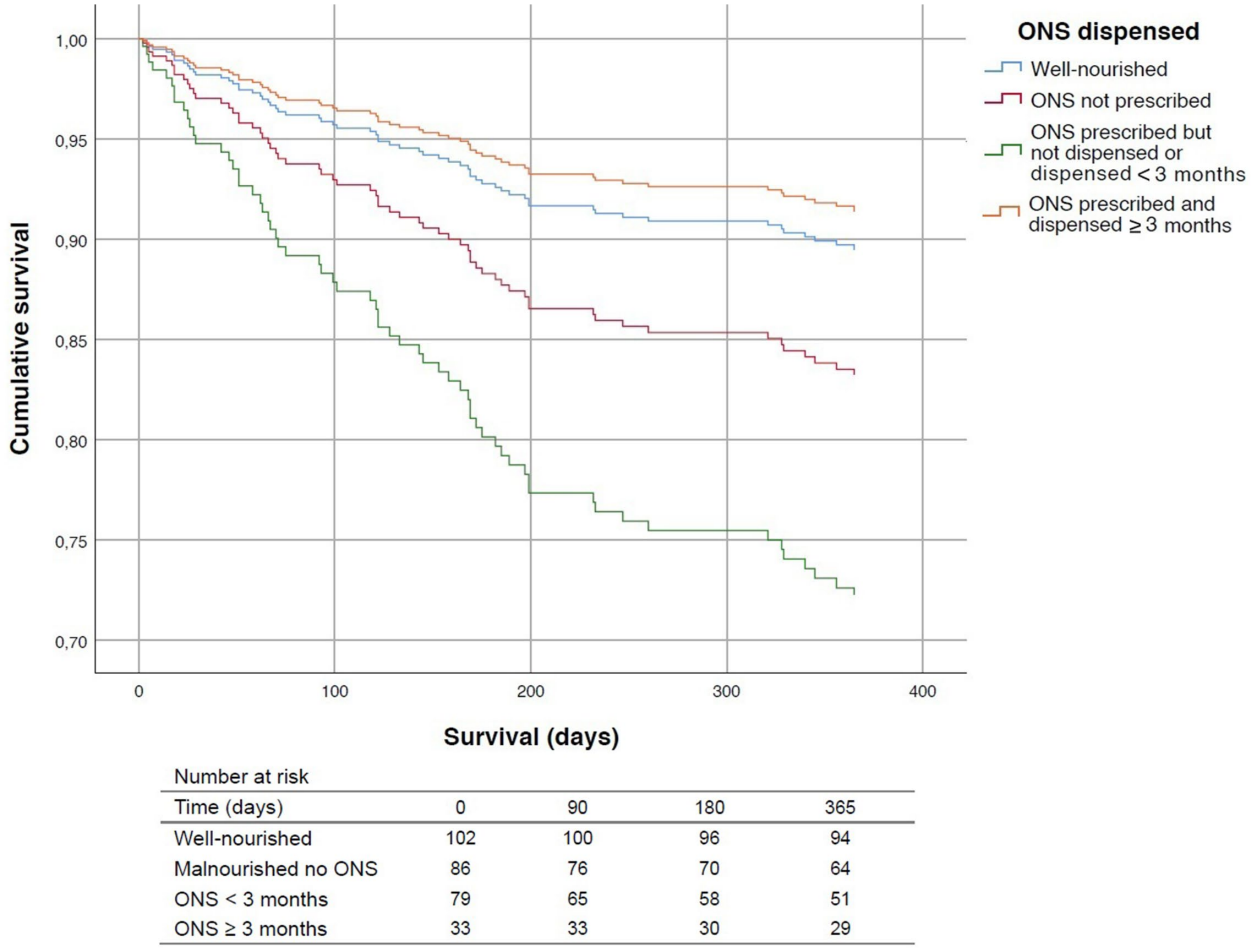
Adjusted survival curves derived from the multivariable Cox regression model according to nutritional status and oral nutritional supplementation (ONS) exposure. Numbers at risk (unadjusted) are shown below the curves.

In the adjusted Cox regression analysis—including adjustment for age, sex, comorbidity, and functional status—significant differences in mortality were observed according to nutritional status and the duration of oral nutritional supplementation (Figure 2). Malnourished patients with ONS prescribed but not retrieved or retrieved for shorter than 3 months showed a significantly higher risk of death than malnourished patients who received ONS for 3 months or more (HR = 3.67; 95% CI: 1.41–9.57; *p* = 0.008). Malnourished patients with ONS prescribed but not retrieved or retrieved for shorter than 3 months had a significantly higher risk of death compared to well-nourished individuals without ONS (HR = 2.95; 95% CI: 1.31–6.65; *p* = 0.009). Moderately malnourished patients without ONS prescribed also had a higher mortality risk than well-nourished patients, although this association was not statistically significant (HR = 1.66; 95% CI: 0.72–3.84; *p* = 0.236). In contrast, malnourished patients who received ONS for 3 months or more showed a non-significant trend toward lower mortality than well-nourished individuals (HR = 0.80; 95% CI: 0.25–2.56; *p* = 0.712), suggesting a potential protective effect of sustained nutritional intervention. To address potential immortal-time bias, a sensitivity analysis was conducted excluding patients who died within the first 30 days after hip fracture surgery. Among malnourished patients with ONS prescribed who survived beyond 30 days (*n* = 105), sustained ONS retrieval for ≥3 months remained independently associated with a significantly lower risk of mortality compared with shorter ONS use (HR 0.37; 95% CI 0.14–0.99; *p* = 0.047), after adjustment for age, sex, Charlson comorbidity index, and baseline functional status (Barthel Index and FAC). In the 3-month landmark analysis, effect estimates were attenuated and no statistically significant differences were observed between nutritional strategies, likely reflecting reduced statistical power due to early event exclusion. Additional Kaplan–Meier sensitivity analyses comparing survival according to ONS dispensing are provided in Supplementary Figure 1. Additional Kaplan–Meier sensitivity analyses comparing survival according to baseline nutritional status are provided in Supplementary Figure 2.

## Discussion

The results of our study suggest that adherence to ONS prescribed to people with malnutrition with a fragility hip fracture after discharge associates to a lower mortality along the first year after the fracture when they are retrieved for longer than 3 months. These findings underscore the importance of systematic nutritional assessment and sustained intervention in patients identified as malnourished. The significant increase in mortality observed among malnourished individuals who received ONS for less than 3 months, along with the non-significant trend toward improved survival in those with longer supplementation, suggests that adherence and duration of nutritional support play a critical role in modifying prognosis. Additionally, malnourished patients who maintained ONS for at least 3 months showed no statistically significant differences compared with well-nourished patients, although a numerically lower mortality was observed. These patients were older and had a lower functionality according to scores, reinforcing the potential of nutritional therapy not only to mitigate but possibly reverse the negative prognostic impact of malnutrition. The group of moderately malnourished patients who were not prescribed ONS showed an intermediate mortality risk. The attenuation of the association in the landmark analysis suggests that the survival benefit associated with sustained ONS use may be driven predominantly by early post-discharge mortality, a period characterized by higher vulnerability and event rates.

Nutritional intervention may be cost-effective and has been associated with improved nutritional status and a greater functional recovery (16). Several randomized controlled trials (RCT) have evaluated the impact of nutritional supplementation in hip fracture patients with varied designs and timing of intervention. For instance, in a RCT conducted in Sweden, patients over 60 receiving a brief course of intravenous nutrition followed by 7 days of ONS (initiated within 48 h post-trauma) had fewer complications and lower mortality over 120 days compared with standard hospital meals alone (29). Other studies, including a quasi-experimental study that provided nutritional support for 5 days postoperatively (30) and RCTs from Israel and the Netherlands (31, 32), generally suggest that early-initiated ONS, whether confined to the hospital stay or extended post-discharge, can improve nutritional biomarkers, preserve muscle mass, and even reduce infection rates and pressure ulcers. However, some retrospective analyses have failed to demonstrate significant differences in mortality, readmissions, or revision surgeries (33, 34). A multicenter RCT in a rehabilitation unit found that patients who received ONS preserved BMI and appendicular lean mass (ALM), whereas these decreased in the control group. However, functional measures such as activities of daily living (ADL) recovery did not show significant differences (35).

In comparison, our real-life study focusing on adherence to ONS months after discharge adds important evidence to this field. Whereas many prior trials have predominantly implemented nutritional supplementation in-hospital, with some extending supplementation post-discharge, our main finding is that adherence to ONS for 3 months or longer was independently associated with significantly lower mortality at 3, 6 and 12 months. Our findings complement recent prospective evidence from the FracNut study (36). Both studies share a similar real-world clinical setting and the use of high-calorie, high-protein ONS. However, it is important to note that the FracNut study did not include mortality as an outcome, focusing instead on the improvement of nutritional status and functional measures. In that cohort, adherence was high (82.8% at 12 weeks), leading to significant gains in weight and muscle strength. Our study builds upon these observations by demonstrating that this clinical improvement translates into a tangible survival advantage. By reporting a nearly 63% reduction in mortality risk among adherent patients, we provide a clinical outcome that complements the nutritional and functional recovery documented in similar observational cohorts. A notable difference lies in the assessment of adherence. The FracNut study used self-reported diaries, defining adherence as the consumption of 75% of the prescribed volumes. In contrast, our study utilized pharmacy dispensing records to define adherence. While self-reports can provide granular data on daily intake, they are often susceptible to recall and social desirability bias. By using dispensing data, our study offers an objective measure of treatment persistence, identifying a clear survival gap between those who maintained the treatment for at least 3 months and those who did not. Additionally, a controlled RCT conducted in Spain in patients over 65 years old with a hip fracture studied the effects of 4-month ONS administration, which resulted in significant improvement in phase angle (PhA) and rectus femoris cross-sectional area and circumference compared to placebo. No significant differences were found in hospital length of stay, quality-of-life measures (BI) or comorbidity (CCI); and mortality was not studied (37).

Our results complement earlier meta-analyses that, although they found trends toward reduced mortality and readmission rates (38), did not always reach statistical significance, perhaps due to variations in adherence or supplement dosing. Moreover, evidence from narrative reviews (16, 39) and a 2016 Cochrane review (40) suggests that early nutritional intervention, especially when administered throughout hospitalization and possibly extended after discharge, can mitigate sarcopenia and assist in functional recovery, which are believed to have downstream effects on survival. Some studies have hinted at a dose-dependent effect on outcomes (16), emphasizing that the positive impact of supplementation may be closely tied to the number and continuity of the supplements received. Overall, these findings collectively underscore that while ONS interventions may not uniformly shorten hospital or rehabilitation stays, sustained adherence appears to be a key factor in reducing one-year mortality, thereby supporting the routine incorporation of nutritional support into the management strategy for malnourished hip fracture patients.

These results also underscore the importance of acting over factors influencing adherence to ONS. Across four complementary studies, several common themes emerge. Lester et al. categorized adherence determinants into contextual (e.g., timing and staff involvement), personal (e.g., frailty, sensory decline, motivation), and product-related factors (e.g., taste, volume, texture), emphasizing the importance of tailored strategies for older adults recovering from hip fractures (41). Liljeberg et al., using the WHO framework, identified 59 specific barriers and facilitators across five domains, reinforcing the influence of structured healthcare delivery, patient beliefs, and social support—consistent with Lester’s findings (42). Wang et al. highlighted similar challenges in cancer patients, where emotional and motivational barriers (e.g., fatigue, low perceived benefit) strongly impacted adherence and individualized nutrition plans and multidisciplinary follow-up improved outcomes (43). Complementing these perspectives, Leon-Sanz et al. conducted a randomized crossover trial in outpatients with disease-related malnutrition and found that compliance was slightly higher and product waste lower when using low-volume, energy-dense ONS (2.4 kcal/mL), particularly when administered in the initial treatment phase—suggesting that early exposure to more concentrated formulations may reduce intake fatigue over time (44). Taken together, these studies underscore that improving ONS adherence requires coordinated, personalized strategies that address sensory tolerability, behavioral motivation, and health system integration, particularly in populations at nutritional risk.

Our findings align with prior studies emphasizing the critical role of nutritional interventions in improving outcomes for hip fracture patients and underscore the importance of sustained ONS use post-discharge. While causality cannot be inferred due to the observational nature of the study, these results are consistent with the hypothesis that malnutrition is a modifiable risk factor when addressed promptly and persistently. They also highlight the clinical relevance of monitoring adherence and continuity of nutritional interventions beyond discharge or initial prescription. Future research, ideally with prospective or randomized designs, should explore the optimal duration, composition, and delivery strategies for ONS to maximize survival and functional recovery in this vulnerable population.

In our adjusted model, classical predictors such as comorbidity burden remained independently associated with mortality, as expected. However, the fact that a nutritional variable—specifically, the continuation of ONS—was also associated with significant differences in survival adds weight to the argument that nutritional interventions deserve a more prominent role in comprehensive post-fracture care strategies. A key strength of this study is the use of robust real life prospective and one-year follow-up data and well-established nutritional assessments.

Our study has several limitations. First, its observational nature and the use of pharmacy dispensing records as a proxy for ONS adherence may not capture actual consumption or dietary patterns, as noted by the lack of quantitative data on total oral caloric intake, which may lead to some degree of misclassification bias. However, in our healthcare environment, pharmacy records are the only reliable means of tracking ONS access at a population level. Furthermore, any such misclassification would likely lead to an underestimation of the treatment effect; the fact that we found a significant 3.67-fold increase in mortality risk among non-adherent patients—even with this indirect measure—strongly suggests a robust clinical impact of ONS on survival. Second, quantitative follow-up of spontaneous oral dietary intake was not performed. However, we hypothesized that ONS adherence acts not only as a nutritional intervention but also as a marker of continuity of care. Sustained adherence likely reflects a patient’s ongoing engagement with the nutritional plan and overall medical recommendations during the vulnerable post-fracture period. Third, we lacked specific data on perioperative and early postoperative indicators—such as blood transfusion requirements, surgical complications, or institutionalization after discharge. These factors are important because they may influence early mortality and act as barriers to long-term ONS adherence. Although we adjusted for baseline comorbidity burden (CCI) and functional status (BI and FAC) to mitigate confounding, the absence of these perioperative variables should be considered when interpreting our findings. Fourth, we did not account for socioeconomic status as a potential confounder. Although the public and universal nature of our healthcare system reduces the impact of economic barriers on treatment access, we cannot rule out that other unmeasured social factors might have influenced clinical outcomes and adherence. Fifth, while some secondary analyses showed trends that did not reach formal statistical significance, the primary multivariable model demonstrates a robust association between ONS adherence and reduced mortality. The consistency of the Hazard Ratios across different groups suggests a protective trend, although we acknowledge that a larger sample size might be required to confirm these secondary findings with greater statistical power.

## Conclusion

Our study suggests that 3-month adherence to ONS is associated with a reduction in 3, 6 and 12-month mortality in older patients with a hip fracture when compared to non-adherent patients and shows a trend toward an improved survival rate than that of well-nourished patients. While these results are encouraging, they should be interpreted within the context of an observational study, and further research is needed to confirm these trends in broader clinical settings. Integrating adherence-focused strategies into discharge planning is critical for improving recovery and survival outcomes. Future prospective studies should validate these findings and explore interventions to enhance adherence.

## Data Availability

The datasets presented in this article are not readily available due to the high degree of clinical specificity and the elderly population studied, the dataset contains indirect identifiers that could lead to patient re-identification. According to our Data Protection Officer (DPO), total anonymization that meets current GDPR standards is not feasible without compromising the scientific integrity of the data. Therefore, the data are restricted and cannot be shared publicly or upon request. Requests to access the datasets should be directed to Luis Ignacio Navas Vela, luis.navas.vela@gmail.com.
