# Supplementary figures and images for: 3-month oral nutritional supplementation adherence impacts positively on survival in malnourished older patients following hip fracture: a real-life study
Source: Front Nutr. 2026 Mar 11;13:1757193. doi: 10.3389/fnut.2026.1757193 (PMC13015789; doi:10.3389/fnut.2026.1757193)

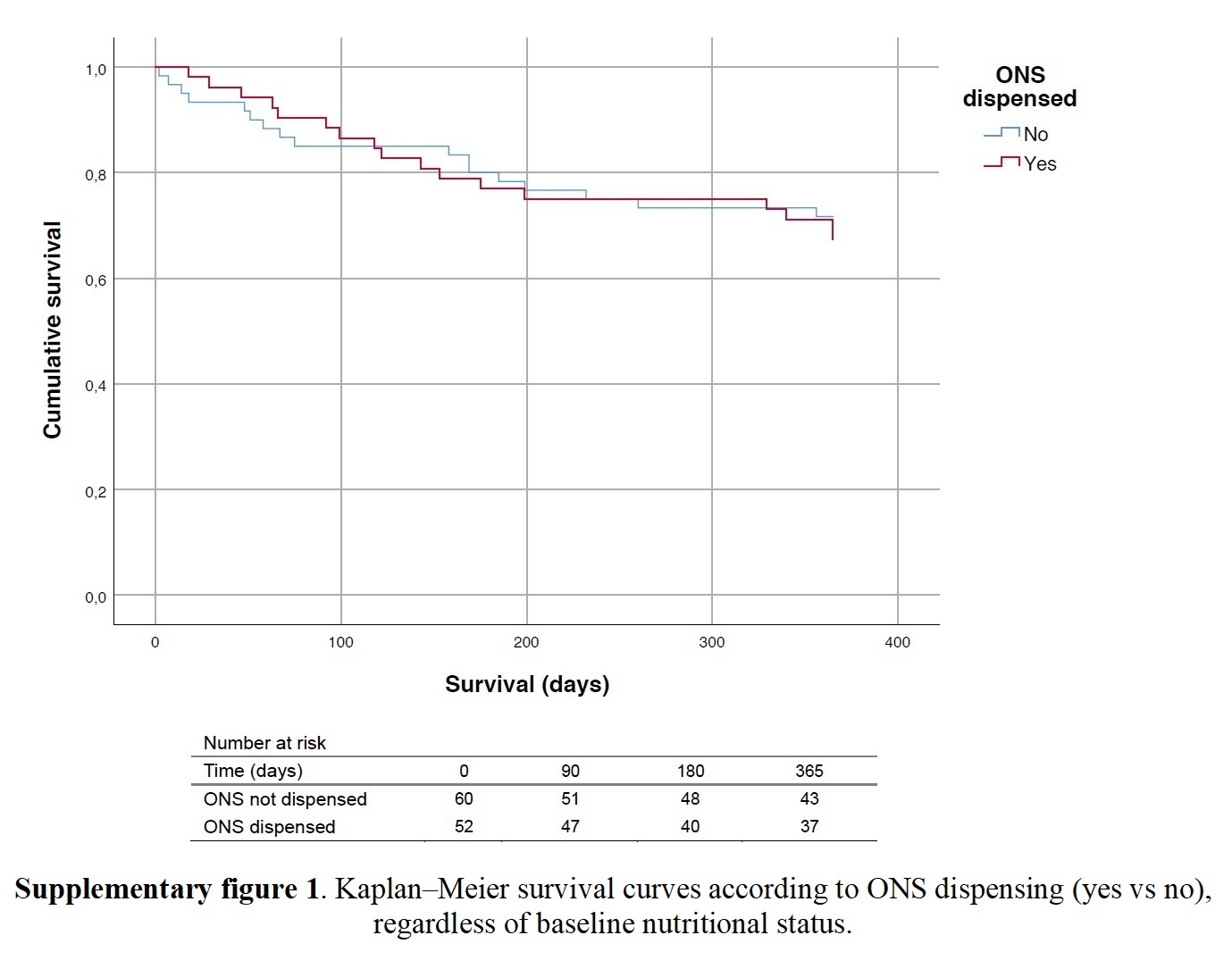

Supplement: Supplementary file 1 [file Image_1.JPEG]

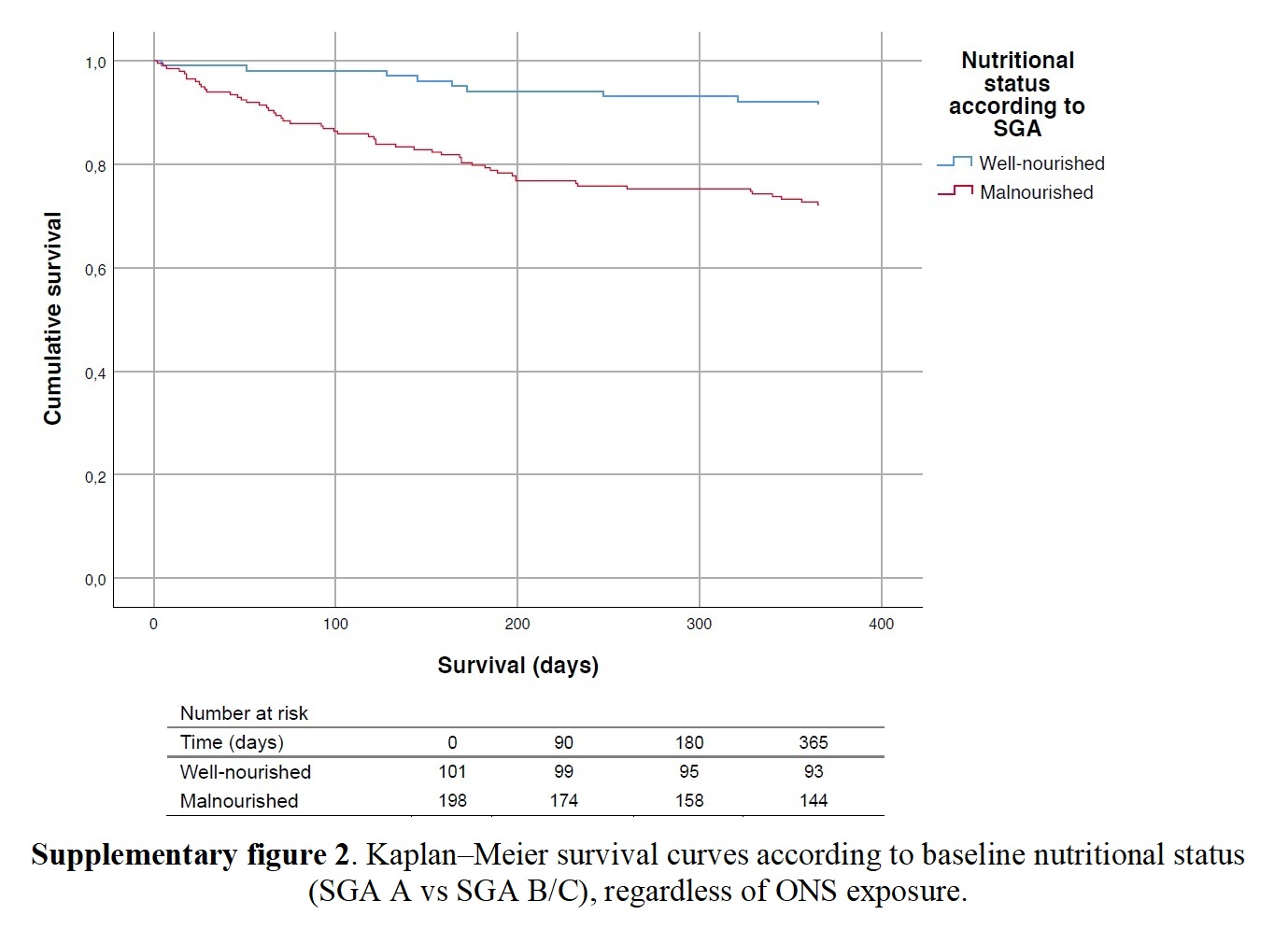

Supplement: Supplementary file 2 [file Image_2.JPEG]
