# Supplementary material for: 3-month oral nutritional supplementation adherence impacts positively on survival in malnourished older patients following hip fracture: a real-life study
Source: Front Nutr. 2026 Mar 11;13:1757193. doi: 10.3389/fnut.2026.1757193 (PMC13015789; doi:10.3389/fnut.2026.1757193)
Supplement: Supplementary file 3 [file Table_1.docx]

Supplementary Material

| **Supplementary table 1. Crude ONS adherence rates according to baseline functional status (Barthel Index categories)** | | | |
| --- | --- | --- | --- |
| Barthel category | Total, n | ONS <3 months, n (%) | ONS ≥3 months, n (%) |
| Independent | 36 | 29 (80.6%) | 7 (19.4%) |
| Mild–moderate dependency | 45 | 30 (66.7%) | 15 (33.3%) |
| Moderate–severe dependency | 31 | 20 (64.5%) | 11 (35.5%) |
| Total | 112 | 79 (70.5%) | 33 (29.5%) |
