# Supplementary material for: 3-month oral nutritional supplementation adherence impacts positively on survival in malnourished older patients following hip fracture: a real-life study
Source: Front Nutr. 2026 Mar 11;13:1757193. doi: 10.3389/fnut.2026.1757193 (PMC13015789; doi:10.3389/fnut.2026.1757193)
Supplement: Supplementary file 4 [file Table_2.docx]

Supplementary Material

| **Supplementary table 2. Crude ONS adherence rates according to baseline comorbidity burden (Charlson Comorbidity Index categories)*** | | | |
| --- | --- | --- | --- |
| Charlson Comorbidity Index category | Total, n | ONS <3 months, n (%) | ONS ≥3 months, n (%) |
| Low–moderate comorbidity | 6 | 6 (100%) | 0 (0%) |
| High comorbidity | 106 | 73 (68.9%) | 33 (31.1%) |
| Total | 112 | 79 (70.5%) | 33 (29.5%) |
| *Most patients presented a high comorbidity burden, with limited representation of lower Charlson categories. | | | |
